# Association of current hepatitis B virus infection with mortality in adults with sepsis

**DOI:** 10.1017/S0950268823000729

**Published:** 2023-05-19

**Authors:** Chang Gao, Jingjing Ni, Ye Gao, Dan Xie, Lijuan Yang, Bining Yang, Xiaoting Lu, Qiang Guo

**Affiliations:** 1 Department of Emergency and Critical Care Medicine, Suzhou Dushu Lake Hospital (Dushu Lake Hospital Affiliated to Soochow University), Suzhou, China; 2 Medical Center of Soochow University, Suzhou, China; 3 Institute of Critical Care Medicine, Soochow University, Suzhou, China; 4 Department of Critical Care Medicine, Zhangjiagang Hospital Affiliated to Soochow University, Suzhou, China; 5 Department of Critical Care Medicine, Taicang Hospital Affiliated to Soochow University, Suzhou, China; 6 Department of Emergency and Critical Care Medicine, Kunshan Hospital of Traditional Chinese Medicine, Suzhou, China; 7 Suzhou Medical College, Soochow University, Suzhou, China; 8 Department of Critical Care Medicine, The First Affiliated Hospital of Soochow University, Suzhou, China

**Keywords:** Current hepatitis B virus (HBV) infection, mortality, sepsis, adults, risk factor

## Abstract

This study aimed to determine the impact of current hepatitis B virus (HBV) infection on patients hospitalised with sepsis. This was a retrospective cohort study. Patients from three medical centres in Suzhou from 10 January 2016 to 23 July 2022 participated in this study. Demographic characteristics and clinical characteristics were collected. A total of 945 adult patients with sepsis were included. The median age was 66.0 years, 68.6% were male, 13.1% presented with current HBV infection, and 34.9% of all patients died. In the multivariable-adjusted Cox model, patients with current HBV infection had significantly higher mortality than those without (hazard ratio (HR) 1.50, 95% confidence interval (CI) 1.11–2.02). A subgroup analysis showed that being infected with HBV significantly increased in-hospital mortality in patients younger than 65 years old (HR 1.74, 95% CI 1.16–2.63), whereas no significant impact was observed in patients ≥65 years. The propensity score-matched case–control analysis showed that the rate of septic shock (91.4% vs. 62.1%, *P* < 0.001) and in-hospital mortality (48.3% vs. 35.3%, *P* = 0.045) were much higher in the propensity score-matched HBV infection group compared with the control group. In conclusion, current HBV infection was associated with mortality in adults with sepsis.

## Introduction

Sepsis, a disease with multiple organ function involvement and a high fatality rate caused by infection, is still one of the severe challenges of global public health events [1, 2]. A nationwide prospective and sectional study showed that sepsis affected one-fifth of patients in the Chinese mainland, with a 90-day mortality of 35.5% and an in-hospital mortality of 51.94% in patients with septic shock [3]. Risk factors for death in sepsis patients included older age, increased sequential organ failure assessment (SOFA) scores, immunosuppression, high level of lactate, and comorbidities [3, 4]. Recent studies showed that, in China, the burden of hospitalization for sepsis is larger than previously estimated [5]. Exploring the risk factors leading to poor prognosis is of great significance for reducing the burden of disease and early identification of critically ill patients.

Hepatitis B is a major global public health problem, and despite impressive progress in preventing chronic infections and reducing mortality, hepatitis B virus (HBV) infection remains a leading cause of premature death [6]. Chronic HBV infection was prone to coinfection with pathogens including bacteria and viruses [7–10]. In disease states of chronic viral infection, persistent T-cell receptors and inflammatory signalling drive CD8^+^ T cells into a state of dysfunction, leading to CD8^+^ T-cell depletion [11]. In addition, T-cell depletion was one of the manifestations of sepsis immunosuppression [12–14]. Some studies have shown that complicating chronic hepatitis B was associated with poor prognosis in coronavirus disease 2019 (COVID-19) patients [10, 15]. Bacterial infection (BI) was associated with high levels of HBV replication [7], and in patients with HBV-related acute-on-chronic liver failure, BI can worsen liver failure and lead to adverse clinical outcomes [16].

However, the effect of coinfection with HBV on the prognosis of sepsis has been poorly described. In this study, compared with patients without HBV infection, septic patients with HBV infection had higher proportions of moderate-to-severe ARDS, AKI, and septic shock. Multivariate analysis showed that coinfected with HBV was an independent risk factor for mortality of sepsis.

## Methods

### Study design and participants

This was a retrospective cohort study. Three medical centres (First Affiliated Hospital of Soochow University, Dushu Lake Hospital Affiliated to Soochow University, and Zhangjiagang Hospital Affiliated to Soochow University) participated in this study. A total of 945 patients who were diagnosed with sepsis from 10 January 2016 to 23 July 2022 were screened for inclusion in the study. Patients who were more than 48 hours from the onset of illness to admission and who died within 48 hours after admission were not included in this study.

The diagnosis of sepsis and septic shock were according to the ‘Third International Consensus Definitions for Sepsis and Septic Shock’ [17]. Sepsis is defined as life-threatening organ dysfunction caused by a dysregulated host response to infection. Organ dysfunction can be identified as an acute change in total SOFA score ≥2 points consequent to the infection. Septic shock is identified with a clinical construct of sepsis with persisting hypotension requiring vasopressors to maintain mean arterial pressure ≥65 mmHg and having a serum lactate level >2 mmol/L (18 mg/dL) despite adequate volume resuscitation [17]. Patients with current HBV infection were defined by HBsAg positivity and/or by ICD-10-CM diagnosis codes [18]. Acute kidney injury was defined as one of the following: an increase in serum creatinine by ≥0.3 mg/dl (≥26.5 μmol/l) within 48 hours; an increase in serum creatinine to ≥1.5 times baseline within the previous 7 days; and urine volume ≤0.5 ml/kg/h for 6 hours. The moderate-to-severe acute respiratory distress syndrome (ARDS) was defined according to the ‘Berlin Definition’, PaO_2_/FiO_2_ ≤100 mm Hg with PEEP ≥5 cmH_2_O [19].

### Data collection

Medical records were reviewed by trained physicians. Demographic characteristics (age and sex) and clinical characteristics (comorbidities, laboratory findings, severity of illness scores, treatments, complications, and outcomes) were collected. For any discrepancies between the two datasets, the original medical records were checked to make sure the data accuracy.

Patients were followed-up from admission to hospital discharge or death (whichever came first). The primary outcome was in-hospital mortality. Secondary outcomes were rates of moderate-to-severe ARDS, AKI, and septic shock.

### Statistical analysis

Continuous data that showed a skewed distribution were presented as median (interquartile range). Frequency data were expressed as proportions. Comparisons of continuous variables were made with the Mann–Whitney *U* test, whereas differences in categorical variables were assessed using the *χ*^2^ test, as appropriate.

Multivariate Cox regression models were used to determine the independent risk factors for death during hospitalization. Variables (without HBV infection) with *P* < 0.1 in univariate Cox proportional hazard regression (Supplementary Table S1) were included in the multivariate analysis. The probabilities of entering and removing variables in a stepwise manner in the multivariate model were 0.05 and 0.10, respectively. To determine the association between HBV infection and mortality, hazard ratios (HRs) and 95% confidence intervals (Cis) were estimated with adjustment of independent risk factors, which are shown in Supplementary Tables S2.

Propensity score matching (one-to-one) between the patients infected with HBV (HBV group) and those who were not (control group) was performed to minimise the selection bias effect of HBV infection. Multiple logistic regressions without considering the outcomes were used to determine propensity scores. Each patient in the HBV group was sought to match with those who had a propensity score that was identical to five digits in the control group. If this is not possible, the algorithm will match four-, three-, two-, or one-digit in turn. Subjects infected with HBV were excluded from the matched analysis if they did not match any of the subjects in the control group. The model’s discrimination was assessed by using C-statistics. We used the Wilcoxon signed-rank test for continuous variables and the McNemar test for binary categorical variables to assess the balance of baseline covariance between the two groups [20].

Data were analysed using SPSS 25.0 (IBM, Chicago, IL, USA) and Stata 16.0 (StataCorp LLC, College Station, TX, USA). A two-tailed *P*-value of <0.05 was considered statistically significant.

### Study approval

This study was approved by the institutional review boards at the First Affiliated Hospital of Soochow University (2019-050), Dushu Lake Hospital Affiliated to Soochow University (2020-20003), and Zhangjiagang Hospital Affiliated to Soochow University (ZJGYYLL-2022-03-001).

## Results

A total of 945 adult inpatients (≥18 years old) with sepsis were admitted to the First Affiliated Hospital of Soochow University, Dushu Lake Hospital Affiliated to Soochow University, and Zhangjiagang Hospital Affiliated to Soochow University from 10 January 2016 to 23 July 2022.

### Patient characteristics

The median age was 66.0 years, 648 (68.6%) were male, hypertension (30.4%) was the most common pre-existing condition, and 224 (23.7%) had diabetes. All patients enrolled in this study completed HBV antigen testing; 124 (13.1%) patients were infected with HBV. Among the 945 patients, 578 (61.2%) presented with the complication of moderate-to-severe ARDS, 552 (58.4%) patients had septic shock, 314 (33.2%) patients presented with AKI, and 330 (34.9%) patients died from infection or comorbidities (Table 1).

**Table 1.** Clinical characteristics of 945 patients with sepsis

Characteristics	All patients (*n* = 945)	Survival (*n* = 615)	Deceased (*n* = 330)	*P*
Age, median (IQR), yr	66.0 (52.0–75.0)	65.0 (50.0–74.0)	67.0 (55.0–77.0)	0.001
Sex, male patients, *n* (%)	648 (68.6)	419 (68.1)	229 (69.4)	0.690
Pre-existing condition, *n* (%)				
Hypertension	287 (30.4)	179 (29.1)	108 (32.7)	0.248
Cardiovascular disease	82 (8.7)	50 (8.1)	32 (9.7)	0.451
Cerebrovascular disease	127 (13.4)	61 (9.9)	66 (20.09)	<0.001
Diabetes	224 (23.7)	144 (23.4)	80 (24.2)	0.775
Infected with HBV	124 (13.1)	63 (10.2)	61 (18.5)	<0.001
Current smokers, *n* (%)	214 (22.6)	134 (21.8)	80 (24.2)	0.390
Alcohol, *n* (%)	137 (14.5)	92 (15.0)	45 (13.6)	0.582
Laboratory findings on admission, median (IQR)				
WBC count, × 10^9^/L	10.7 (6.6–17.2)	10.9 (6.8–17.4)	10.3 (6.1–16.9)	0.192
Platelet count, × 10^9^/L	150.0 (87.0–223.0)	157.0 (99.0–234.0)	131.0 (66.0–183.0)	<0.001
ALT, U/L	38.3 (18.1–92.2)	39.3 (17.9–89.9)	35.9 (−97.7)	0.917
AST, U/L	46.0 (23.4–116.7)	43.5 (22.6–104.3)	52.0 (25.3–144.0)	0.023
BUN, mmol/L	10.5 (6.6–17.6)	9.9 (6.2–16.0)	12.5 (7.5–19.8)	<0.001
Serum creatinine, μmol/L	101.3 (61.0–179.3)	96.0 (61.0–163.8)	113.1 (60.7–193.4)	0.063
Lactic acid, mmol/L	2.6 (1.5–4.3)	2.2 (1.3–3.6)	3.3 (1.9–5.3)	<0.001
APACHE II, median (IQR)	14.0 (10.0–19.0)	13.0 (9.0–18.0)	16.0 (11.0–22.0)	<0.001
SOFA, median (IQR)	7.0 (5.0–9.0)	7.0 (4.0–9.0)	8.0 (6.0–10.0)	<0.001
Treatments and Complications, *n* (%)				
Use of MV	587 (62.1)	322 (52.4)	265 (80.3)	<0.001
Use of CRRT	154 (16.3)	77 (12.5)	77 (23.3)	<0.001
Moderate-to-severe ARDS	578 (61.2)	326 (53.0)	252 (76.4)	<0.001
AKI	314 (33.2)	183 (29.8)	131 (39.7)	0.002
Septic shock	552 (58.4)	342 (55.6)	210 (63.6)	0.017

Abbreviations: AKI, acute kidney injury; ALT, alanine transaminase; APACHE II, acute physiology and chronic health II; ARDS, acute respiratory distress syndrome; AST, aspartate aminotransferase; BUN, blood urea nitrogen; CRRT, continuous renal replacement therapy; HBV, hepatitis B virus; IQR, interquartile range; MV, mechanical ventilation; SOFA, sequential organ failure assessment; WBC, white blood cell.

Of all the patients, pneumonia was the most common initial infection source and abdominal infections rank the second. In the HBV group, 41.1% of patients suffered from pneumonia, 24.2% presented with gastrointestinal tract infection, 16.1% presented with hepatobiliary system infection, and 5.6% presented with blood infection, whereas the rates were 57.1%, 13.3%, 5.8%, and 2.2% previously in patients without HBV (Table 2).

**Table 2.** Initial infection sources of 945 patients with sepsis

Initial infection sources	All patients (*n* = 945)	HBV (*n* = 124)	Non-HBV (*n* = 821)
Lung	520 (55.0)	51 (41.1)	469 (57.1)
Gastrointestinal tract	139 (14.7)	30 (24.2)	109 (13.3)
Hepatobiliary system	68 (7.2)	20 (16.1)	48 (5.8)
Pancreas	80 (8.5)	9 (7.3)	71 (8.6)
Urinary tract system	48 (5.1)	2 (1.6)	46 (5.6)
Blood	25 (2.6)	7 (5.6)	18 (2.2)
Others	65 (6.9)	5 (4.0)	60 (7.3)

Compared with the survival group, patients in the deceased group presented with higher median values of blood urea nitrogen (12.5 vs. 9.9 mmol/L, *P* < 0.001), lactic acid (3.3 vs. 2.2 mmol/L, *P* < 0.001), aspartate aminotransferase (52.0 vs. 43.5 U/L, *P* = 0.023), APACHE II score (16.0 vs. 13.0, *P* < 0.001), and SOFA score (7.0 vs. 8.0, *P* < 0.001) at the time of admission, but lower median values of platelet counts (131.0 vs. 157.0 × 10^9^/L, *P* < 0.001) (Table 1).

**Figure 1.**
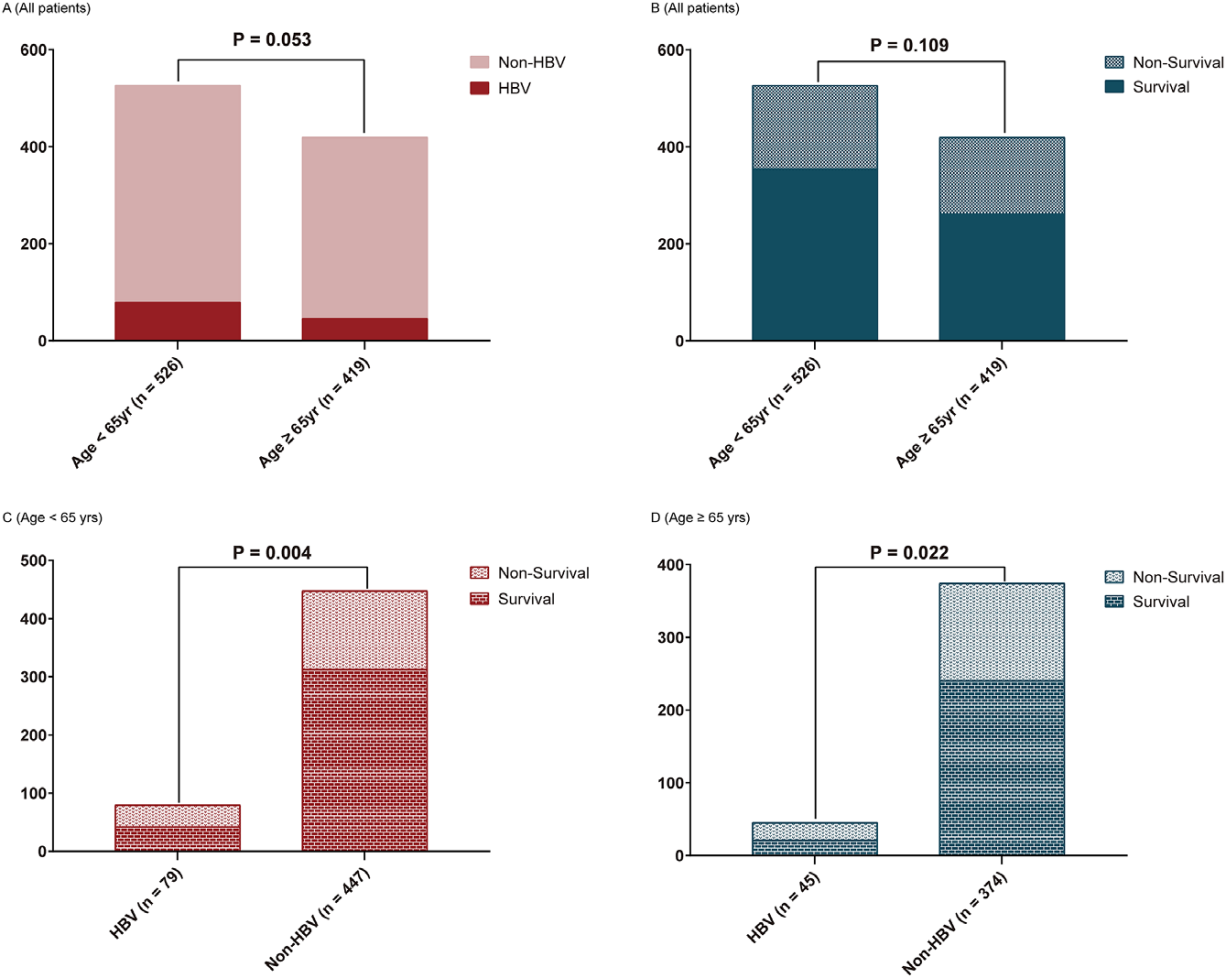
The difference in clinical outcomes in different ages and HBV groups.

Higher proportions of the deceased group required mechanical ventilation (80.3% vs. 52.4%, *P* < 0.001) and continuous renal replacement therapy (23.2% vs. 12.5%, *P* < 0.001) than the survival group. Compared with the survival group, more patients were infected with HBV in the deceased group (54.6% vs. 44.2%, *P* = 0.030) (Table 1). In both subgroups younger than 65 years of age and equal or older, mortality was significantly higher in the HBV infection group than in the control group (Figure 1).

### Association between HBV infection and in-hospital mortality

**Figure 2.**
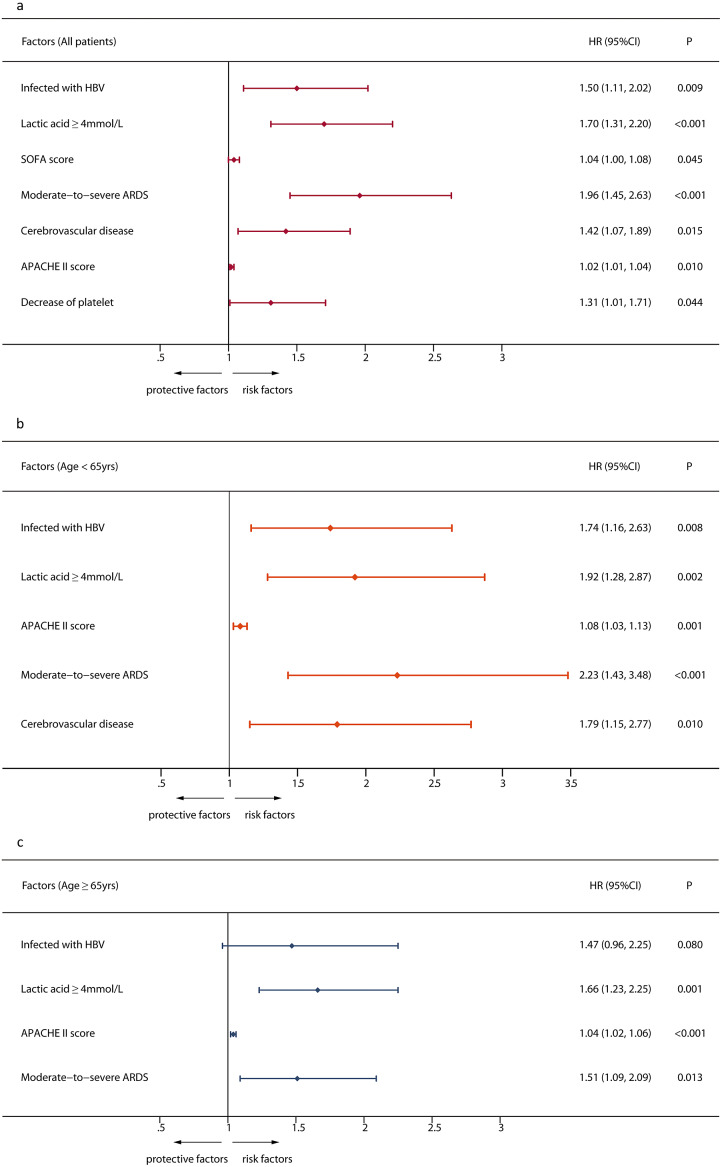
Multivariate Cox regression analysis of current HBV infection associated with mortality in adult septic inpatients. a. Association of current HBV infection and mortality in all patients. b. Association of current HBV infection and mortality in subgroup of patients < 65 years old. c. Association of current HBV infection and mortality in subgroup of patients ≥ 65 years old.

The univariate Cox regression analysis (Supplementary Table S1) determined that with the exception of HBV infection, many other factors including pre-existing conditions, laboratory findings, and complications were associated with in-hospital mortality. In the multivariable-adjusted Cox proportional hazard regression model (adjustment of independent risk factors including cerebrovascular disease, lactic acid ≥4 mmol/L, decrease of platelet, moderate-to-severe ARDS, SOFA score, and APACHE II score showed in Supplementary Tables S2), patients who infected with HBV had a significantly higher in-hospital mortality than those in the control group (HR 1.50, 95% CI 1.11–2.02) (Figure 2a). A subgroup analysis showed that being infected with HBV significantly increased in-hospital mortality in patients younger than 65 years old (HR 1.74, 95% CI 1.16–2.63) (Figure 2b), whereas no significant impact was observed in patients ≥65 years (Figure 2c).

### Impact of HBV infection on in-hospital mortality using propensity score-matched case–control analysis

We used a multivariate logistic regression model to calculate the propensity score. The C-statistics was 0.74. The HBV infection and control groups generated a total of 116 propensity score matching pairs. Baseline characteristics (including age, sex, pre-existing conditions, laboratory findings, and critical illness scores) between HBV infection with matching propensity scores and control groups were similar (Table 3). The rate of septic shock (91.4% vs. 62.1%, *P* < 0.001) and in-hospital mortality (48.3% vs. 35.3%, *P* = 0.045) were much higher in the propensity score-matched HBV infection group compared with the control group. No significant difference was found in the rates of AKI and moderate-to-severe ARDS between the two propensity score-matched groups (Table 3).

**Table 3.** Baseline and outcomes of sepsis patients in the unmatched and matched HBV and control groups

Characteristics	Unmatched groups			Matched groups		
	HBV (*n* = 124)	Control (*n* = 821)	*P* ^a^	HBV (*n* = 116)	Control (*n* = 116)	*P* ^b^
Age, median (IQR), yr	58.5 (45.0–69.8)	66.0 (52.0–76.0)	<0.001	57.5 (44.3–68.8)	60.0 (45.0–69.8)	0.930
Sex, male patients, *n* (%)	91 (73.4)	557 (67.8)	0.215	84 (72.4)	73 (62.9)	0.161
Pre-existing condition, *n* (%)						
Hypertension	35 (28.2)	252 (30.7)	0.577	31 (26.7)	28 (24.1)	0.766
Cardiovascular disease^c^	5 (4.0)	77 (9.4)	0.049	5 (4.3)	6 (5.2)	1.000
Cerebrovascular disease^d^	20 (16.1)	107 (13.0)	0.346	19 (16.4)	14 (12.1)	0.486
Diabetes	20 (16.1)	204 (24.8)	0.033	20 (17.2)	19 (16.4)	1.000
Current smokers, *n* (%)	25 (20.2)	189 (23.0)	0.478	24 (20.7)	15 (12.9)	0.188
Alcohol, *n* (%)	16 (12.9)	121 (14.7)	0.589	15 (12.9)	14 (12.1)	1.000
Laboratory findings on admission, median (IQR)						
WBC count, × 10^9^/L	9.5 (5.0–18.6)	10.8 (6.7–17.1)	0.403	9.5 (5.0–17.9)	11.5 (5.6–16.6)	0.573
Platelet count, × 10^9^/L	102.0 (51.0–161.0)	154.0 (94.0–229.0)	<0.001	109.5 (55.0–171.3)	126.5 (64.0–185.8)	0.060
ALT, U/L	42.1 (18.1–97.1)	38.1 (18.1–92.1)	0.467	42.1 (18.0–97.1)	38.3 (20.0–72.2)	0.227
AST, U/L	48.0 (22.3–100.2)	45.8 (24.0–119.2)	0.705	48.0 (22.3–100.2)	51.5 (29.8–125.1)	0.281
BUN, mmol/L	9.1 (5.6–15.9)	10.8 (6.7–17.7)	0.060	8.9 (5.3–16.0)	11.8 (6.6–17.8)	0.101
Serum creatinine, μmol/L	95.0 (61.0–153.0)	103.7 (61.0–183.0)	0.279	93.0 (60.0–153.0)	128.5 (65.7–194.4)	0.063
Lactic acid, mmol/L	3.4 (2.0–5.4)	2.4 (1.4–4.1)	<0.001	3.4 (2.0–5.4)	3.1 (1.8–5.1)	0.902
APACHE II, median (IQR)	16.5 (12.3–22.0)	14.0 (10.0–18.0)	<0.001	16.0 (12.0–22.0)	17.0 (13.0–23.0)	0.255
SOFA, median (IQR)	6.5 (4.0–10.0)	7.0 (5.0–9.0)	0.380	7.0 (4.0–10.0)	7.5 (5.0–9.8)	0.639
Treatments and complications, *n* (%)						
Use of MV	63 (50.8)	524 (63.8)	0.005	59 (50.9)	76 (65.5)	0.041
CRRT	26 (21.0)	128 (15.6)	0.131	25 (21.6)	21 (18.1)	0.626
Moderate-to-severe ARDS^e^	61 (49.2)	517 (63.0)	0.003	57 (49.1)	69 (59.5)	0.142
AKI	57 (46.0)	257 (31.3)	0.001	54 (46.6)	47 (40.5)	0.391
Septic shock	114 (91.9)	438 (53.3)	<0.001	106 (91.4)	72 (62.1)	<0.001
Outcomes, *n* (%)						
In-hospital mortality	61 (49.2)	269 (32.8)	<0.001	56 (48.3)	41 (35.3)	0.045

Abbreviations: AKI, acute kidney injury; ALT, alanine transaminase; APACHE II, acute physiology and chronic health II; ARDS, acute respiratory distress syndrome; AST, aspartate aminotransferase; BUN, blood urea nitrogen; CRRT, continuous renal replacement therapy; HBV, hepatitis B virus; IQR, interquartile range; MV, mechanical ventilation; SOFA, sequential organ failure assessment; WBC, white blood cell.

a The Mann–Whitney *U* test was used for the continuous variables, and the chi-square test was used for the categorical variables.

b The Wilcoxon signed-rank test was used for the continuous variables, and the McNemar test was used for the categorical variables.

c Cardiovascular disease was defined as congestive heart failure, known conduction system abnormality, or ischemic heart disease.

d Cerebrovascular disease was defined as intracerebral haemorrhage and ischemic strokes.

e Moderate-to-severe ARDS was diagnosed according to the Berlin definition: PaO_2_/FiO_2_ ratio of ≤200 mmHg and a positive end-expiratory pressure of ≥5 cmH_2_O.

## Discussion

HBV is one of the infectious diseases with the largest number of patients and clinical significance in the world, and there are a large number of hepatitis B patients in China [7]. Sepsis is one of the most common serious complications caused by infectious diseases, which is a very important public health problem with a high fatality rate [17, 21]. Previous studies have shown that coinfection of hepatitis B patients with other viruses may be associated with poorer clinical course [10, 15, 22]. It is not clear whether there is a possibility of worse outcomes after sepsis in patients with hepatitis B infection. In this study, the incidence of septic shock and in-hospital mortality were higher in patients infected with HBV than those without. HBV infection was independently associated with in-hospital mortality in septic patients. The mortality rate for sepsis in our cohort was 34.9%, which is similar to the mortality rate for septic shock (32.6%–41.5%) and sepsis (21.5%–37.5%) shown in previous studies [23].

Studies have shown that HBV and other viruses such as severe acute respiratory syndrome coronavirus 2 (SARS-CoV-2) and human immunodeficiency virus coinfection can lead to a higher risk of death [8, 15]. Higher rates of liver injury in patients coinfected with SARS-CoV-2 and HBV were associated with increased rates of shock and cardiac injury, which may lead to poor prognosis. [15, 24]. Hepatitis B patients were prone to BI, and the DNA level of patients with BI was significantly elevated [7]. BI in patients with chronic hepatitis may lead to decompensation of liver cirrhosis and increase the risk of infection with drug-resistant bacteria [25]. BI may be a trigger for gastrointestinal bleeding, ascites, hepatic encephalopathy, and renal failure, even if it occurs during the compensatory period of liver function [25, 26]. In addition, antimicrobial agents and vasoactive agents used in patients with HBV infection during the treatment of sepsis or septic shock may increase the incidence of drug-induced liver injury and thus aggravate the disease [27].

Chronic HBV infection has been shown to be associated with virus-specific CD4^+^ and CD8^+^ T-cell failure because of the persistence of viral antigens [11, 28, 29]. Sepsis is also associated with T-cell depletion, and sepsis-associated CD4^+^ T-cell and CD8^+^ T-cell apoptosis led to lymphocytopenia, immunosuppression, and increased susceptibility to secondary infections in patients with advanced sepsis [14]. The CD4^+^ cells decimated lead to a decreased Th17 cytokine response and thus an increased susceptibility to fungal infections, which may negatively affect mortality. [12, 13, 30]. Sepsis with HBV infection may exacerbate these changes and thus increase immunosuppression, which may be associated with higher mortality.

Thrombocytopenia is common in patients with sepsis and septic shock and is associated with worse outcomes [1]. Pro-coagulant upregulation leads to platelet depletion and clotting factor depletion, leading to classic sepsis-related thrombocytopenia [1]. In HBV patients, autoimmunity is an important factor leading to thrombocytopenia, and T-cells immunity plays an important role in autoimmunity [31]. We found that the platelet counts in the HBV group were significantly lower than those in the non-HBV group, which may partly explain the association between HBV infection and higher mortality in septic patients.

Sepsis and sepsis-related mortality increased significantly with age [32–34]. Old age (≥65 years old) is one of the high-risk factors for immunosuppression in septic patients [35]. Based on this, a subgroup analysis was conducted in this study. Results showed that HBV infection remained an independent risk factor for death in patients with sepsis at age <65 years; however, no similar results were seen in the older patients (age ≥ 65 years) subgroup. This may be related to higher disease severity in older patients with sepsis. Moreover, the present study found that other independent risk factors for death in septic patients included moderate-to-severe ARDS, elevated lactic acid, and elevated critical illness score, consistent with previous studies [1, 36].

Due to the limitations of its retrospective nature, this study lacks baseline levels of HBV DNA, and patients could not be grouped according to the HBV infection phases. We did not have access to basic liver involvement and cytokine changes in patients with HBV infection. This study included only three medical centres, and larger studies are needed to verify the impact of current HBV infection on sepsis. The mechanism of interaction between HBV infection and sepsis needs to be further explored.

## Conclusion

Current HBV infection was associated with mortality in adults with sepsis. Sepsis with HBV infection should be considered, especially in patients under 65 years of age.

## Data Availability

The data that support the findings of this study are available from the corresponding author upon reasonable request.

## Supplementary material

For supplementary material accompanying this paper visit http://doi.org/10.1017/S0950268823000729. click here to view supplementary material
