# Supplementary material for: Association of current hepatitis B virus infection with mortality in adults with sepsis
Source: Epidemiol Infect. 2023 May 19;151:e94. doi: 10.1017/S0950268823000729 (PMC10311682; doi:10.1017/S0950268823000729)
Supplement: Supplementary file 1 [file S0950268823000729sup001.docx]

Appendix

**Association of current hepatitis B virus infection with mortality in adults with sepsis**

**Table S1. Univariate Cox regression analysis of factors associated with mortality in severe COVID-19 adult patients.**

| **Factors** | **Unadjusted HR (95% CI)** | ***P*** |
| --- | --- | --- |
| Age ≥ 65 yr | 1.05 (0.84-1.30) | 0.686 |
| Male sex | 0.91 (0.72-1.15) | 0.419 |
| Current smokers | 1.10 (0.86-1.42) | 0.439 |
| Alcohol | 0.95 (0.70-1.31) | 0.7642 |
| Hypertension *^a^* | 1.20 (0.95-1.51) | 0.118 |
| Diabetes *^a^* | 1.04 (0.81-1.33) | 0.783 |
| Cardiovascular disease *^a^* | 1.04 (0.72-1.49) | 0.847 |
| Cerebrovascular [disease](javascript:;) *^a^* | 1.77 (1.35-2.32) | <0.001 |
| Current HBV infection | 1.78 (1.34-2.35) | <0.001 |
| White blood cell counts | 1.01 (0.99-1.01) | 0.115 |
| Aminotransferase *^b^* | 1.00 (1.00-1.00) | 0.043 |
| Aspartate aminotransferase *^b^* | 1.00 (1.00-1.00) | 0.004 |
| Blood urea nitrogen *^b^* | 1.02 (1.01-1.04) | <0.001 |
| Serum creatinine *^b^* |  |  |
| Lactate ≥ 4mmol/l *^b^* | 2.22 (1.78-2.76) | <0.001 |
| APACHE II score | 1.05 (1.04-1.07) | <0.001 |
| SOFA score | 1.11 (1.07-1.14) | <0.001 |
| Decrease of platelet | 1.67 (1.33-2.08) | <0.001 |
| Moderate to severe ARDS | 2.00 (1.55-2.58) | <0.001 |
| AKI | 1.38 (1.10-1.72) | 0.005 |

*^a^* Pre-existing condition.

*^b^* Laboratory findings on admission.

Abbreviations: HBV, hepatitis B virus; APACHE II, acute physiological and chronic health II; SOFA, sequential organ failure assessment; ARDS, acute respiratory distress syndrome; AKI, acute kidney injury; HR, Hazard ratio; CI, confidence interval.

**Table S2. Multivariate Cox regression analysis of factors associated with mortality in septic adult patients.**

| **Subgroups** | **Adjusted HR (95% CI)** | ***P*** |
| --- | --- | --- |
| **All patients** |  |  |
| Cerebrovascular [disease](javascript:;) *^a^* | 1.43 (1.08-1.90) | 0.014 |
| Decrease of platelet *^b^* | 1.36 (1.04-1.77) | 0.023 |
| Lactate ≥ 4mmol/l *^b^* | 1.77 (1.37-2.30) | <0.001 |
| Moderate to severe ARDS | 1.86 (1.38-2.49) | <0.001 |
| APACHE II score | 1.03 (1.01-1.05) | 0.003 |
| SOFA score | 1.04 (1.00-1.07) | 0.044 |
| **Age < 65 years** |  |  |
| Cerebrovascular [disease](javascript:;) *^a^* | 1.59 (1.11-2.26) | 0.011 |
| Lactate ≥ 4mmol/l | 1.82 (1.28-2.57) | 0.001 |
| Moderate to severe ARDS | 1.92 (1.32-2.78) | 0.001 |
| APACHE II score | 1.04 (1.01-1.06) | 0.001 |
| **Age ≥ 65 years** |  |  |
| Lactate ≥ 4mmol/l | 1.86 (1.33-2.60) | <0.001 |
| Moderate to severe ARDS | 1.49 (1.01-2.18) | 0.043 |
| APACHE II score | 1.04 (1.02-1.07) | 0.001 |

*^a^* Pre-existing condition.

*^b^* Laboratory findings on admission.

The Cox regression analysis included the variables listed in Table S1 (without current HBV infection).

Result of current HBV infection associated with mortality was shown in figure 2.

Abbreviations: ARDS, acute respiratory distress syndrome; APACHE II, acute physiological and chronic health II; SOFA, sequential organ failure assessment; HR, Hazard ratio; CI, confidence interval
